# Supplementary material for: Association of MALAT1 and PVT1 Variants, Expression Profiles and Target miRNA-101 and miRNA-186 with Colorectal Cancer: Correlation with Epithelial-Mesenchymal Transition
Source: Int J Mol Sci. 2021 Jun 7;22(11):6147. doi: 10.3390/ijms22116147 (PMC8201273; doi:10.3390/ijms22116147)
Supplement: Supplementary file 1 [file ijms-22-06147-s001.zip › ijms-1247563-supplementary.pdf]

Supplementary Material

Table S1: Minor allele frequencies of the studied SNPs

	MAF in Our Studied Population	Global MAF$	Highest Population MAF$
**MALAT1.rs3200401**	T = 0.34	T = 0.14	T = 0.31
**PVT1.rs13255292**	T = 0.42	T = 0.20	T = 0.43

MAF, minor allele frequency. $according to Ensembl release 102 - November 2020 ©.

Table S2: Hardey-Winberg equilibrium of rs3200401 and rs13255292in CRC group

**MALAT.rs3200401.C.T. Exact Test for Hardy-Weinberg Equilibrium (*n* = 280)**						
	**CC**	**CT**	**TT**	**C**	**T**	***p* Value**
**All subjects**	73	110	57	256	224	0.24
**CONTROL**	42	48	10	132	68	0.66
**CRC**	31	62	47	124	156	0.23
**PVT.rs13255292.C.T. Exact Test for Hardy-Weinberg Equilibrium (*n* = 280)**						
	**CC**	**CT**	**TT**	**C**	**T**	***p* Value**
**All subjects**	95	117	28	307	173	0.4
**CONTROL**	31	55	14	117	83	0.22
**CRC**	64	62	14	190	90	1

Hardey-Winberg equilibrium was done by chi-square test. $p < 0.05$ means statistical significance. AIC: Akaike's Information Criteria, BIC: Bayesian Information Criteria.

Table S3: *MALAT1 rs3200401 CT* association with control and AP groups

***MALAT1 rs3200401 CT* Association with Control and AP Groups (*n* = 140, Adjusted by AGE+GENDER)**							
**Model**	**Genotype**	**CONTROL**	**AP**	**OR (95% CI)**	**P-value**	**AIC**	**BIC**
Codominant	C/C	42 (42%)	17 (42.5%)	1.00	0.26	167	181.7
	C/T	48 (48%)	15 (37.5%)	0.94 (0.41–2.20)			
	T/T	10 (10%)	8 (20%)	2.37 (0.76–7.37)			
Dominant	C/C	42 (42%)	17 (42.5%)	1.00	0.65	167.5	179.2
	C/T-T/T	58 (58%)	23 (57.5%)	1.20 (0.55-2.60)			
Recessive	C/C-C/T	90 (90%)	32 (80%)	1.00	0.1	165	176.7
	T/T	10 (10%)	8 (20%)	2.44 (0.85–6.98)			
Overdominant	C/C-T/T	52 (52%)	25 (62.5%)	1.00	0.48	167.2	178.9
	C/T	48 (48%)	15 (37.5%)	0.76 (0.35–1.64)			
Log-additive	---	---	---	1.38 (0.80–2.41)	0.25	166.3	178.1

SNPs association was done using SNPstat software analysis. $p < 0.05$ means statistical significance. AIC: Akaike's Information Criteria, BIC: Bayesian Information Criteria.

Table S4: *PVT1 rs13255292 CT* association with control and AP groups

PVT1 rs13255292 CT Association with Control and AP Groups(*n* = 140, Adjusted by AGE+GENDER)							
**Model**	**Genotype**	**CONTROL**	**AP**	**OR (95% CI)**	**P-value**	**AIC**	**BIC**
Codominant	CC	31 (31%)	16 (40%)	1.00	0.7	169	183.7
	CT	55 (55%)	20 (50%)	0.79 (0.35–1.79)			
	TT	14 (14%)	4 (10%)	0.60 (0.16–2.20)			
Dominant	CC	31 (31%)	16 (40%)	1.00	0.47	167.1	178.9
	CT-TT	69 (69%)	24 (60%)	0.75 (0.34–1.65)			
Recessive	CC-CT	86 (86%)	36 (90%)	1.00	0.53	167.3	179
	TT	14 (14%)	4 (10%)	0.69 (0.20–2.31)			
Overdominant	CC-TT	45 (45%)	20 (50%)	1.00	0.78	167.6	179.4
	CT	55 (55%)	20 (50%)	0.90 (0.42–1.92)			
Log-additive	---	---	---	0.78 (0.43–1.40)	0.4	167	178.7

SNPs association was done using SNPstat software analysis. $p < 0.05$ means statistical significance. AIC: Akaike's Information Criteria, BIC: Bayesian Information Criteria.

Table S5: Correlation between the studied SNPs and clinicopathological factors

MALAT1 rs3200401 CT Association with Response Anatominal Site (*n* = 140, Adjusted by AGE+Age)								*PVT1 rs13255292 CT* Association with Response Anatominal Site (*n* = 140, Adjusted by AGE+Age)							
**Model**	**Genotype**	**Rectum**	**Colon**	**OR (95% CI)**	***p* Value**	**AIC**	**BIC**	**Model**	**Genotype**	**Rectum**	**Colon**	**OR (95% CI)**	***p* Value**	**AIC**	**BIC**
Codominant	T/T	11 (29.7%)	36 (35%)	1	0.54	168.1	179.8	Codominant	C/C	14 (37.8%)	50 (48.5%)	1	0.27	166.6	178.4
	C/T	19 (51.4%)	43 (41.8%)	0.69 (0.29–1.63)					C/T	17 (46%)	45 (43.7%)	0.72 (0.32–1.64)			
	C/C	7 (18.9%)	24 (23.3%)	1.12 (0.38–3.36)					T/T	6 (16.2%)	8 (7.8%)	0.36 (0.11–1.23)			
Dominant	T/T	11 (29.7%)	36 (35%)	1	0.59	167	175.8	Dominant	C/C	14 (37.8%)	50 (48.5%)	1	0.24	165.9	174.7
	C/T-C/C	26 (70.3%)	67 (65%)	0.80 (0.35–1.81)					C/T-T/T	23 (62.2%)	53 (51.5%)	0.63 (0.29–1.36)			
Recessive	T/T-C/T	30 (81.1%)	79 (76.7%)	1	0.48	166.8	175.6	Recessive	C/C-C/T	31 (83.8%)	95 (92.2%)	1	0.15	165.3	174.1
	C/C	7 (18.9%)	24 (23.3%)	1.40 (0.54–3.66)					T/T	6 (16.2%)	8 (7.8%)	0.43 (0.14–1.34)			
Overdominant	T/T-C/C	18 (48.6%)	60 (58.2%)	1	0.28	166.1	174.9	Overdominant	C/C-T/T	20 (54%)	58 (56.3%)	1	0.78	167.2	176
	C/T	19 (51.4%)	43 (41.8%)	0.66 (0.31–1.40)					C/T	17 (46%)	45 (43.7%)	0.90 (0.42–1.91)			

Log-Additive	---	---	---	1.01 (0.60–1.69)	0.97	167.3	176.1	Log-Additive	---	---	---	0.63 (0.36–1.12)	0.12	164.8	173.7

MALAT1 rs3200401 CT **Association with Stage (*n* = 140, Adjusted by AGE+Age)**								***PVT1 rs13255292 CT*** **Association with Stage (*n* = 140, Adjusted by AGE+Age)**							
**Model**	**Genotype**	**Stage I+II**	**Stage= III +IV**	**OR (95% CI)**	***p* value**	**AIC**	**BIC**	**Model**	**Genotype**	**Stage I+II**	**Stage= III +IV**	**OR (95% CI)**	***p* Value**	**AIC**	**BIC**
Codominant	T/T	32 (36.4%)	15 (28.9%)	1	0.33	189.9	201.7	Codominant	C/C	38 (43.2%)	26 (50%)	1	0.76	191.6	203.4
	C/T	35 (39.8%)	27 (51.9%)	1.66 (0.75–3.68)					C/T	41 (46.6%)	21 (40.4%)	0.76 (0.37–1.58)			
	C/C	21 (23.9%)	10 (19.2%)	0.94 (0.35–2.53)					T/T	9 (10.2%)	5 (9.6%)	0.83 (0.25–2.76)			
Dominant	T/T	32 (36.4%)	15 (28.9%)	1	0.38	189.4	198.2	Dominant	C/C	38 (43.2%)	26 (50%)	1	0.47	189.6	198.5
	C/T-C/C	56 (63.6%)	37 (71.2%)	1.39 (0.66–2.92)					C/T-T/T	50 (56.8%)	26 (50%)	0.77 (0.39–1.54)			
Recessive	T/T-C/T	67 (76.1%)	42 (80.8%)	1	0.42	189.5	198.3	Recessive	C/C-C/T	79 (89.8%)	47 (90.4%)	1	0.92	190.1	199
	C/C	21 (23.9%)	10 (19.2%)	0.70 (0.30–1.67)					T/T	9 (10.2%)	5 (9.6%)	0.94 (0.30–2.99)			
Overdominant	T/T-C/C	53 (60.2%)	25 (48.1%)	1	0.14	187.9	196.8	Overdominant	C/C-T/T	47 (53.4%)	31 (59.6%)	1	0.5	189.7	198.5
	C/T	35 (39.8%)	27 (51.9%)	1.70 (0.84–3.42)					C/T	41 (46.6%)	21 (40.4%)	0.79 (0.39–1.58)			
Log-Additive	---	---	---	1.03 (0.64–1.64)	0.91	190.1	199	Log-Additive	---	---	---	0.85 (0.50–1.45)	0.55	189.8	198.6

**MALAT1 rs3200401 CT Association with Response Lymphnode Status (*n* = 140, Adjusted by AGE+Age)**								***PVT1 rs13255292 CT*** **Association with Response Lymphnode Status (*n* = 140, Adjusted by AGE+Age)**							
**Model**	**Genotype**	**Absent**	**Present**	**OR (95% CI)**	***p* Value**	**AIC**	**BIC**	**Model**	**Genotype**	**Absent**	**Present**	**OR (95% CI)**	***p* Value**	**AIC**	**BIC**
Codominant	T/T	25 (28.4%)	22 (42.3%)	1	0.065	186.9	198.7	Codominant	C/C	38 (43.2%)	26 (50%)	1	0.75	191.8	203.6
	C/T	39 (44.3%)	23 (44.2%)	0.67 (0.31–1.46)					C/T	41 (46.6%)	21 (40.4%)	0.76 (0.37–1.57)			
	C/C	24 (27.3%)	7 (13.5%)	**0.30 (0.11–0.86)**					T/T	9 (10.2%)	5 (9.6%)	0.82 (0.25–2.74)			
Dominant	T/T	25 (28.4%)	22 (42.3%)	1	0.086	187.5	196.3	Dominant	C/C	38 (43.2%)	26 (50%)	1	0.46	189.9	198.7
	C/T-C/C	63 (71.6%)	30 (57.7%)	0.53 (0.26–1.09)					C/T-T/T	50 (56.8%)	26 (50%)	0.77 (0.39–1.54)			
Recessive	T/T-C/T	64 (72.7%)	45 (86.5%)	1	0.035	185.9	194.8	Recessive	C/C-C/T	79 (89.8%)	47 (90.4%)	1	0.92	190.4	199.2

	C/C	24 (27.3%)	7 (13.5%)	**0.38 (0.15–0.98)**					T/T	9 (10.2%)	5 (9.6%)	0.94 (0.30–2.98)			
Overdominant	T/T-C/C	49 (55.7%)	29 (55.8%)	1	0.96	190.4	199.2	Overdominant	C/C-T/T	47 (53.4%)	31 (59.6%)	1	0.5	189.9	198.8
	C/T	39 (44.3%)	23 (44.2%)	1.02 (0.51–2.04)					C/T	41 (46.6%)	21 (40.4%)	0.79 (0.39–1.58)			
Log-Additive	---	---	---	**0.57 (0.35–0.93)**	0.023	185.2	194	Log-Additive	---	---	---	0.85 (0.50–1.44)	0.54	190	198.8

***MALAT1 rs3200401 CT* Association with Response Metastasis (*n* = 140, adjusted by AGE+Age)**								***PVT rs13255292 CT* Association with Response Metastasis (*n* = 140, Adjusted by AGE+Age)**							
**Model**	**Genotype**	**Absent**	**Present**	**OR (95% CI)**	***p* Value**	**AIC**	**BIC**	**Model**	**Genotype**	**Absent**	**Present**	**OR (95% CI)**	***p* Value**	**AIC**	**BIC**
Codominant	T/T	36 (31%)	11 (45.8%)	1	0.35	133.8	145.5	Codominant	C/C	52 (44.8%)	12 (50%)	1	0.91	135.7	147.4
	C/T	53 (45.7%)	9 (37.5%)	0.56 (0.21–1.49)					C/T	52 (44.8%)	10 (41.7%)	0.85 (0.34–2.15)			
	C/C	27 (23.3%)	4 (16.7%)	0.45 (0.13–1.59)					T/T	12 (10.3%)	2 (8.3%)	0.74 (0.15–3.76)			
Dominant	T/T	36 (31%)	11 (45.8%)	1	0.16	131.9	140.7	Dominant	C/C	52 (44.8%)	12 (50%)	1	0.68	133.7	142.5
	C/T-C/C	80 (69%)	13 (54.2%)	0.52 (0.21–1.28)					C/T-T/T	64 (55.2%)	12 (50%)	0.83 (0.34–2.01)			
Recessive	T/T-C/T	89 (76.7%)	20 (83.3%)	1	0.39	133.1	142	Recessive	C/C-C/T	104 (89.7%)	22 (91.7%)	1	0.77	133.8	142.6
	C/C	27 (23.3%)	4 (16.7%)	0.60 (0.19–1.96)					T/T	12 (10.3%)	2 (8.3%)	0.80 (0.17–3.82)			
Overdominant	T/T-C/C	63 (54.3%)	15 (62.5%)	1	0.5	133.4	142.3	Overdominant	C/C-T/T	64 (55.2%)	14 (58.3%)	1	0.81	133.8	142.6
	C/T	53 (45.7%)	9 (37.5%)	0.73 (0.30–1.82)					C/T	52 (44.8%)	10 (41.7%)	0.90 (0.37–2.19)			
Log-Additive	---	---	---	0.64 (0.35–1.20)	0.16	131.9	140.7	Log-additive	---	---	---	0.86 (0.43–1.70)	0.66	133.7	14

Correlation was done using SNPstat software analysis. $p < 0.05$ means statistical significance. AIC: Akaike's Information Criteria, BIC: Bayesian Information Criteria.

Table S6: Correlation between the studied parameters and clinicopathological factors

**Spearman Correlation**	**MALAT1**	**miRNA-101**	**PVT1**	**miRNA-186**	**E-Cadherin**	**Age**	**Gender**	**Anatominal Site**	**Stage**	**Lymphnode Status**	**Metastasis**
MALAT1	1.0000	−0.4025	0.3608	−0.3208	−0.3236	0.0953	0.1020	0.1925	0.3340	0.3600	0.3062
miRNA-101	−0.4025	1.0000	0.0132	0.0238	0.3559	−0.0532	0.0071	0.0185	−0.4132	−0.3390	-0.3377
PVT1	0.3608	0.0132	1.0000	−0.4688	−0.3078	0.1436	0.1685	−0.1660	0.4412	0.3181	0.3018
miRNA-186	−0.3208	0.0238	−0.4688	1.0000	0.5002	−0.0440	−0.1413	−0.0266	−0.2976	−0.1790	−0.4011

E-cadherin	−0.1947	0.2302	−0.1831	0.1299	1.0000	0.0247	0.2519	−0.1096	−0.1618	−0.2078	−0.3953
Age	0.0953	−0.0532	0.1436	−0.0440	−0.1060	1.0000	−0.2072	−0.0601	0.0760	0.0545	0.0760
Gender	0.1020	0.0071	0.1685	−0.1413	0.0751	−0.2072	1.0000	−0.1280	0.0132	0.0132	0.0170
Anatominal site	0.1925	0.0185	−0.1660	−0.0266	0.0388	−0.0601	−0.1280	1.0000	−0.2098	−0.1427	0.0577
Stage	0.3348	−0.4132	0.4412	−0.3976	−0.2878	0.0760	0.0132	−0.2098	1.0000	0.6635	0.3172
Lymphnode status	0.3348	−0.3390	0.3181	−0.2790	−0.2288	0.0545	0.0132	−0.1427	0.6635	1.0000	0.4740
Metastasis	0.3062	−0.3377	0.3018	−0.4611	−0.4483	0.0760	0.0170	0.0577	0.3172	0.4740	1.0000
**Correlations *p* Value**	**MALAT1**	**miRNA-101**	**PVT1**	**miRNA-186**	**E-Cadherin**	**Age**	**Gender**	**Anatominal Site**	**Stage**	**Lymphnode Status**	**Metastasis**
MALAT1		0.0061	0.0149	0.0435	0.0343	0.5285	0.5002	0.2000	0.0231	0.0191	0.0385
miRNA-101	0.0061		0.9323	0.8856	0.0207	0.7287	0.9633	0.9040	0.0048	0.0227	0.0233
PVT1	0.0149	0.9323		0.0019	0.0447	0.3411	0.2629	0.2703	0.0021	0.0312	0.0415
miRNA-186	0.0435	0.8856	0.00199		0.0012	0.7849	0.3780	0.8689	0.0588	0.2627	0.0094
E-cadherin	0.2108	0.1424	0.23418	0.42422		0.8737	0.0991	0.4789	0.2939	0.1759	0.0079
Age	0.5285	0.7287	0.34114	0.78489	0.4882		0.0140	0.4805	0.3724	0.5226	0.3723
Gender	0.5002	0.9633	0.26291	0.37805	0.6238	0.0140		0.1318	0.8768	0.8768	0.8424
Anatominal site	0.2000	0.9040	0.27029	0.86892	0.8000	0.4805	0.1318		0.0129	0.0925	0.4982
Stage	0.0230	0.0048	0.00215	0.05882	0.0552	0.3724	0.8768	0.0129		< 0.0001	< 0.0001
Lymphnode status	0.0230	0.0227	0.03119	0.26269	0.1305	0.5226	0.8768	0.0925	< 0.0001		< 0.0001
Metastasis	0.0385	0.0233	0.04150	0.00935	0.0020	0.3723	0.8424	0.4982	< 0.0001	< 0.0001	

Correlation was done by spearman correlation. $p < 0.05$ means statistical significance.
